# Effect of Cadmium-Tolerant Rhizobacteria on Growth Attributes and Chlorophyll Contents of Bitter Gourd under Cadmium Toxicity

**DOI:** 10.3390/plants9101386

**Published:** 2020-10-17

**Authors:** Muhammad Zafar-ul-Hye, Muhammad Naeem, Subhan Danish, Muhammad Jamil Khan, Shah Fahad, Rahul Datta, Martin Brtnicky, Antonin Kintl, Ghulam Sabir Hussain, Mohamed A. El-Esawi

**Affiliations:** 1 Department of Soil Science, Faculty of Agricultural Sciences and Technology, Bahauddin Zakariya University, Multan 60800, Pakistan; zafarulhye@bzu.edu.pk (M.Z.-u.-H.); ch.naeem276@gmail.com (M.N.); 2 Department of Soil and Environmental Sciences, Gomal University, Dera Ismail Khan 29220, Pakistan; jamil@gu.edu.pk; 3 Hainan Key Laboratory for Sustainable Utilization of Tropical Bioresource, College of Tropical Crops, Hainan University, Haikou 570228, China; 4 Department of Agronomy, The University of Haripur, Haripur 22620, Pakistan; 5 Department of Agrochemistry, Soil Science, Microbiology and Plant Nutrition, Faculty of AgriSciences, Mendel University in Brno, 61300 Brno, Czech Republic; martin.brtnicky@mendelu.cz; 6 Department of Geology and Pedology, Faculty of Forestry and Wood Technology, Mendel University in Brno, Zemedelska 3, 61300 Brno, Czech Republic; 7 Institute of Chemistry and Technology of Environmental Protection, Faculty of Chemistry, Brno University of Technology, 621 00 Brno, Czech Republic; 8 Agricultural Research, Ltd., 664 41 Troubsko, Czech Republic; kintl@vupt.cz; 9 Department of Technical Services, Fatima Agri Sales and Services, Multan 60800, Pakistan; hussainsabirsial90@yahoo.com; 10 Botany Department, Faculty of Science, Tanta University, Tanta 31527, Egypt; mohamed.elesawi@science.tanta.edu.eg

**Keywords:** heavy metal, mineral fertilizers, *Momordica charantia*, pigments, productivity, PGPR

## Abstract

Cadmium (Cd) is one of the heavy metals that negatively affects the growth of plants. High solubilization in water leads Cd to enter into plants quite easily, thus decreasing seed germination, photosynthesis, and transpiration. It also shows an antagonistic effect with many of the plants’ nutrients like Mn, Ca, K, Mg and Fe. Nowadays, inoculation of plants with ACC deaminase (ACCD) rhizobacteria to mitigate Cd’s adverse effects has drawn the attention of environmental microbiologists. The rhizobacteria secrete organic compounds that can immobilize Cd in soil. Therefore, this study was accomplished to investigate the effect of ACCD plant growth promoting rhizobacteria (PGPR) on the bitter gourd under Cd stress. There were six treatments consisting of two ACCD PGPR (*Stenotrophomonas maltophilia* and *Agrobacterium fabrum*) strains and inorganic fertilizers at two levels of Cd, i.e., 2 (Cd2) and 5 mg kg^−1^ soil (Cd5). The results showed *A. fabrum* with the recommended NPK fertilizer (RNPKF) significantly increased the vine length (48 and 55%), fresh weight (24 and 22%), and contents of chlorophyll a (79 and 50%), chlorophyll b (30 and 33%) and total chlorophyll (61 and 36%), over control at the two Cd levels i.e., Cd2 and Cd5, respectively. In conclusion, the recommended NPK fertilizer + *A. fabrum* combination is a very effective treatment with which to immobilize Cd in soil for the improvement of bitter gourd growth.

## 1. Introduction

Bitter gourd (*Momordica charantia*) contains high amounts of vitamins, proteins, and carbohydrates [1]. As a subtropical and tropical vine, it belongs to the family Cucurbitaceae. Bitter gourd is widely cultivated as edible fruit in Africa, Asia and the Caribbean. Its many varieties differ substantially in the shape and bitterness of the fruit. This tendril-bearing and herbaceous vine grows up to 16 fts in length with alternate leaves 1.6–4.7 inches across, with 3 to 7 deeply separated lobes. It bears yellow female and male flowers. In the Northern Hemisphere, flowering occurs during June–July while fruiting occurs during September–November [2,3]. Bitter melon originated in India and was introduced into China in the 14th century. It is the richest source of vitamin C and protein as compared to tomato and cucumber. Out of the total area on which bitter gourd is cultivated in Pakistan (6107 ha), its total production per year is 57,190 T [4]. Research output in recent years has shed light on the multifarious health benefits of bitter gourd seeds, fruits, leaves, and other parts of the plant. These effects encompass antidiabetic, antiobesity, antitumor, antifungal, anti-HIV, and antibacterial activities. Most of the plant parts, especially the seeds, contain oil. Bitter gourd seed oil is rich in stearic acid, oleic acid, and linoleic acid, and it exhibits antidiabetic and antitumor activities. In view of the fact that bitter gourd is a common dietary component and its safety has been demonstrated, studies on bitter melon essential oil are interesting and have promising commercial applications. However, yield of bitter gourd is adversely affected when cultivated in heavy-metals-polluted soil [5].

High use of pesticides and industrial pollution has increased the concentration of heavy metals tremendously. Wastewater of industries has a large amount of As, Cd, Cr, Ni and Pb, and is frequently used as an irrigation source for crop cultivation [6]. The use of organic fertilizers also leads to the risk of xenobiotic contamination [7,8,9,10], Besides which, anthropogenic activities have also enhanced heavy metals contamination in cultivatable areas [6,11]. Cadmium (Cd) is toxic which affects the growth and yield of crops [12,13]. Higher translocation of Cd in plants causes a significant decrease in seed germination, root length, transpiration rate, photosynthetic rate, and plant height [14,15,16]. Also, deficiency of iron under Cd-induced stress causes chlorosis in plants [15,17,18]. It has been observed that Cd also shows an antagonistic relationship with calcium, manganese, magnesium, and potassium [19,20]. Furthermore, imbalance of water, alteration in membrane permeability, and instability of lipid membrane in plants play an imperative role in the reduction of growth and yield attributes in stress conditions [21,22,23].

Stress conditions stimulate the methionine to change into S-adenosyl-Met. Activation of enzyme ACC synthase converts S-adenosyl-Met into ACC. This ACC is further catalyzed by ACC oxidase, which converts it into stress-generating ethylene. Accumulation of stress ethylene in plants decreases the length of the root while increasing its thickness. The thickness in roots due to stress-generating ethylene is mainly characterized by the accumulation of dead cells in the cortex, which result in lysigenous aerenchyma formation. However, poor roots and shoot lengths are characterized by inhibition of cell division due to higher accumulation of ethylene in hypocotyls [24,25,26]. Higher reactive oxygen species (ROS) due to Cd stress is a major drawback that disturbs the electron transport chain in the chloroplast [27]. These ROS also damage the proteins, lipids, and nucleic acid—thus inducing oxidative stress [28].

Inoculation of metals-tolerant plant growth promoting rhizobacteria (PGPR) is gaining importance currently, to overcome the damaging effects of heavy metals [12,29]. Secretions of phytohormones and growth regulators, i.e., siderophores, indole acetic acid, and gibberellins by certain PGPR improve the uptake of nutrients, root length, chlorophyll contents [30,31,32,33]. Inoculation of ACC deaminase (ACCD)-producing PGPR also decreases the stress ethylene by breaking it into ammonia and α-ketobutyrate [12,24,34,35,36,37,38]. In addition, a decrease in the uptake of Cd due to PGPR is a key benefit that is linked with better growth and chlorophyll contents improvement in the crops. Therefore, the current study was conducted with the aim of investigating the effectiveness of ACCD PGPR i.e., *A. fabrum* and *S. maltophilia* with recommended inorganic fertilizer. It is hypothesized that *A. fabrum* and *S. maltophilia* with recommended inorganic fertilizer is an effective approach to improve the vine and root length, fresh weight, and chlorophyll in bitter gourd under Cd toxicity.

## 2. Results

### 2.1. Vine Length

**Figure 1:**
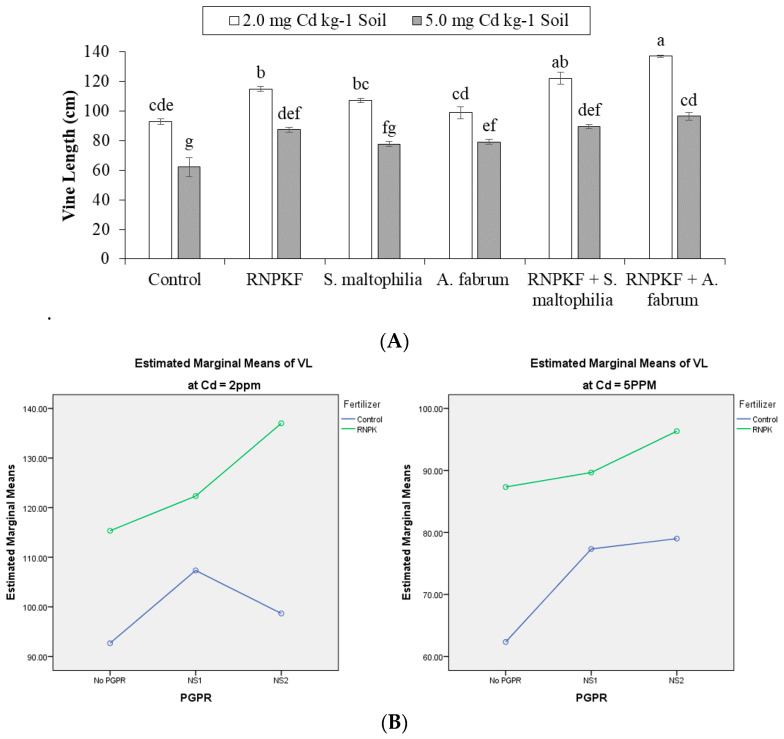

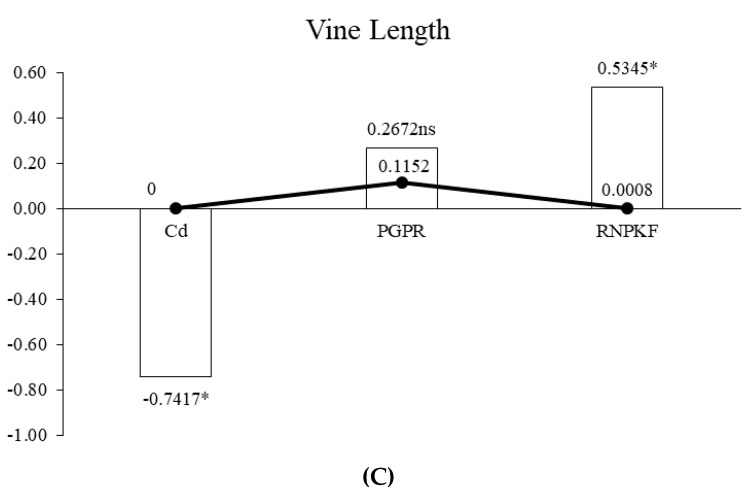
Effect of rhizobacteria with and without recommended NPK fertilizer on vine length (VL) of bitter gourd under Cd (**A**). Interaction graph of PGPR (NS1 = *S. maltophilia*, NS2 = *A. fabrum*), recommended NPK fertilizer (RNPKF) and Cd levels for vine length (cm) (**B**). Pearson correlation of vine length with different levels of Cd, ACC deaminase (ACCD) PGPR and recommended NPK fertilizer where the *y*-axis represents the values of correlation (bars in the graph) and *x*-axis probability (lines in the graph) (**C**). * = *p* ≤ 0.05; ns = *p* > 0.05 non-significant.

Results showed that treatments *A. fabrum*, recommended NPK fertilizer, and *S. maltophilia* significantly (*p* ≤ 0.05) affected the vine length (VL) of bitter gourd plants under Cd2 (2 mg cadmium kg^−1^ soil) and Cd5 (5 mg cadmium kg^−1^ soil). Inoculation of PGPR and recommended NPK fertilizer had significant interactive and main effects on VL at Cd2. Only the main effects (ME) of PGPR and the recommended NPK fertilizer were significant for VL at Cd5 (Figure 1A). PGPR × recommended NPK fertilizer did not differ significantly but was ordinal at Cd5 for vine length (Figure 1B). The treatments recommended: NPK fertilizer + *S. maltophilia;* and recommended NPK fertilizer + *A. fabrum* were significantly different from the control under Cd2 and Cd5. Statistically, similar responses were noted among recommended NPK fertilizer and *S. maltophilia* for vine length, while recommended NPK fertilizer was significantly better than the control for vine length at Cd2. Sole inoculation of *A. fabrum* gave a significant positive response over the control for vine length at Cd5. However, the performance of recommended NPK fertilizer + *A. fabrum* was better than that of *A. fabrum* for the improvement in vine length at Cd2 and Cd5. Correlation of vine length was negatively significant (−0.7417) with Cd2 and Cd5. Non-significantly positive correlation (0.2672) was noted between PGPR and vine length. However, significantly positive (0.5345) correlation was recorded between vine length and recommended NPK fertilizer (Figure 1C). There was a significant increase of 47.8 and 55.0% in vine length with the recommended NPK fertilizer + *A. fabrum* from control at Cd2 and Cd5, respectively.

### 2.2. Root Length

**Figure 2:**
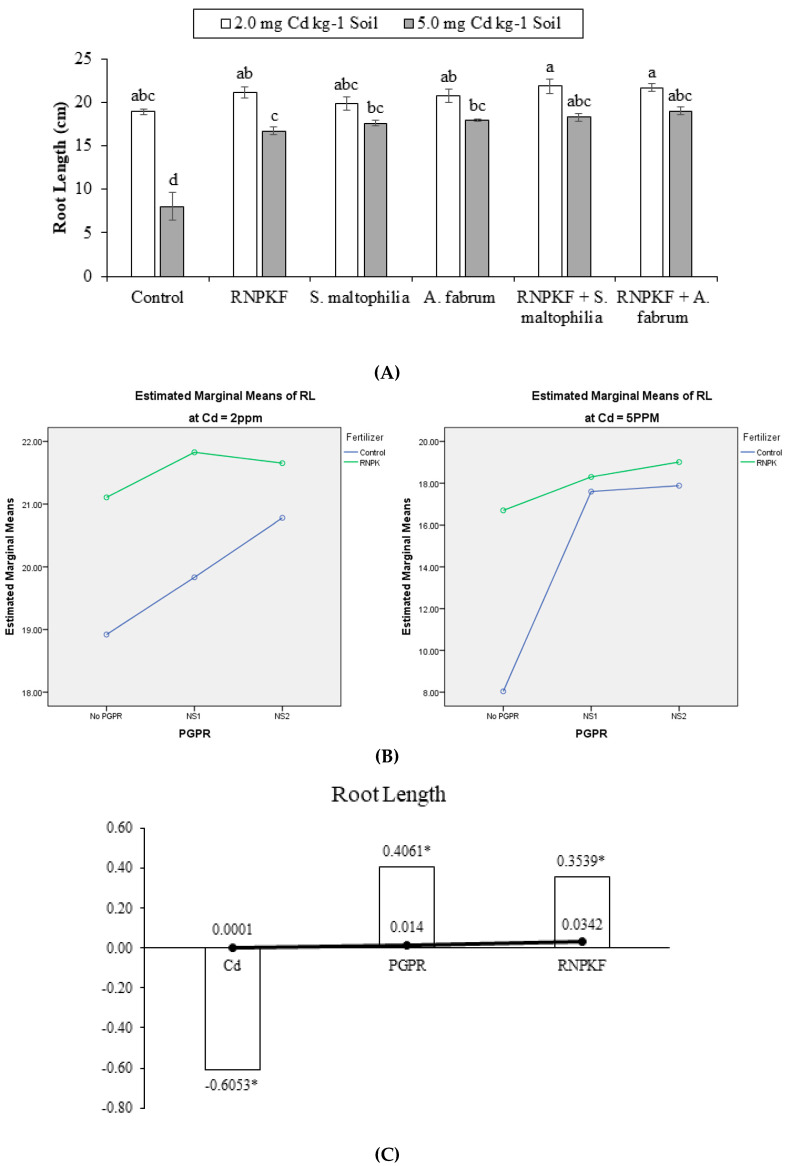
Effect of rhizobacteria with and without recommended NPK fertilizer on root length (RL) of bitter gourd under Cd (**A**). Interaction graph of PGPR (NS1 = *S. maltophilia*, NS2 = *A. fabrum*), RNPKF and Cd levels for root length (cm) (**B**). Pearson correlation of root length with different levels of Cd, ACCD PGPR, and recommended NPK fertilizer where the *y*-axis represents the values of correlation (bars in the graph) and *x*-axis probability (lines in the graph) (**C**). * = *p* ≤ 0.05; ns = *p* > 0.05 non-significant.

Inoculation of *S. maltophilia*, *A. fabrum,* and application of recommended NPK fertilizer under Cd (Cd2 and Cd5) significantly (*p* ≤ 0.05) affected the root length (RL) of bitter gourd plants. Application of recommended NPK fertilizer has a significant ME of PGPR and recommended NPK fertilizer on RL (at Cd2). In the case of Cd5, both interactive and main effects of PGPR and recommended NPK fertilizer were significantly different for RL (Figure 2A). At Cd2, the interactive effect of PGPR × recommended NPK fertilizer was ordinal, while PGPR × recommended NPK fertilizer was significantly ordinal at Cd5 for root length (Figure 2B). All the treatments remained statistically alike to each other for root length at Cd2. Recommended NPK fertilizer + *S. maltophilia*; recommended NPK fertilizer + *A.fabrum*; recommended NPK fertilizer; *S. maltophilia;* and *A. fabrum* differed significantly for root length compared to the control at Cd5 toxicity. Treatments with recommended NPK fertilizer + *S. maltophilia*; recommended NPK fertilizer + *A. fabrum*; recommended NPK fertilizer; *S. maltophilia;* and *A. fabrum* did not differ significantly from each other for root length at Cd5 toxicity. Correlation of root length was negatively significant (−0.6053) at the different levels of Cd. Significantly positive (0.4061) correlation was recorded between PGPR and root length as well as between root length and recommended NPK fertilizer (0.3539) (Figure 2C). Treatment with recommended NPK fertilizer + *A. fabrum* plants showed the highest increase of 136% in root length at Cd5.

### 2.3. Bitter Gourd Fresh Weight

**Figure 3:**
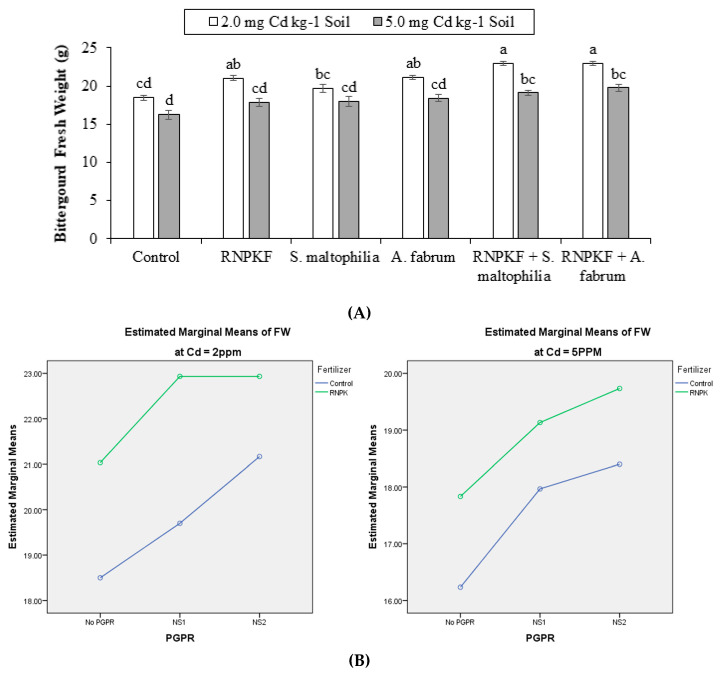

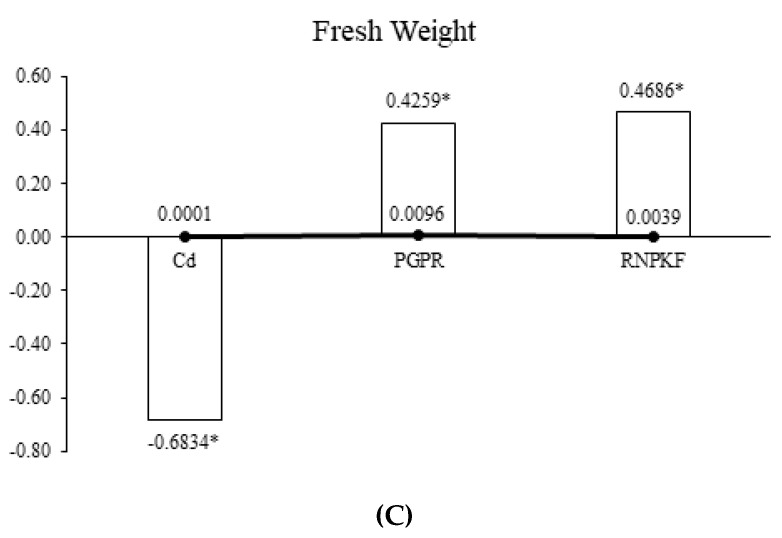
Effect of rhizobacteria with and without recommended NPK fertilizer on fresh weight (FW) of bitter gourd under Cd (**A**). Interaction graph of PGPR (NS1 = *S. maltophilia*, NS2 = *A. fabrum*), RNPKF and Cd levels for fresh weight (cm) (**B**). Pearson correlation of fresh weight with different levels of Cd, ACCD PGPR, and recommended NPK fertilizer where the *y*-axis represents the values of correlation (bars in the graph) and *x*-axis probability (lines in the graph) (**C**).* = *p* ≤ 0.05; ns = *p* > 0.05 non-significant.

Bitter gourd fresh weight (FW) was significantly (*p* ≤ 0.05) affected by *S. maltophilia*, *A. fabrum,* and recommended NPK fertilizer at Cd2 and Cd5. The main effect of PGPR and recommended NPK fertilizer remained significant on the FW at Cd2 and Cd5 but they had non-significant interactions (Figure 3A). Results showed that PGPR × recommended NPK fertilizer interaction was ordinal at Cd2 (Figure 3B) but was disordinal at Cd5 for FW (Figure 3B). Application of recommended NPK fertilizer + *S. maltophilia*, recommended NPK fertilizer + *A. fabrum*, and recommended NPK fertilizer and *A. fabrum* were significantly better than control at Cd2 for FW. In the case of Cd5, recommended NPK fertilizer + *S. maltophilia* and recommended NPK fertilizer + *A. fabrum* showed a significant positive response over the control for the improvement in FW at Cd5. Correlation of fresh weight was negatively significant (−0.6834) at the different levels of Cd. Significantly positive correlation (0.4259) was recorded between PGPR and fresh weight as well as between fresh weight and recommended NPK fertilizer (0.4686) (Figure 3C). Application of recommended NPK fertilizer + *A. fabrum* gave 24% and 22% higher improvements in the FW compared to the control at Cd2 and Cd5, respectively.

### 2.4. Chlorophyll a Content in Bitter Gourd

**Figure 4:**
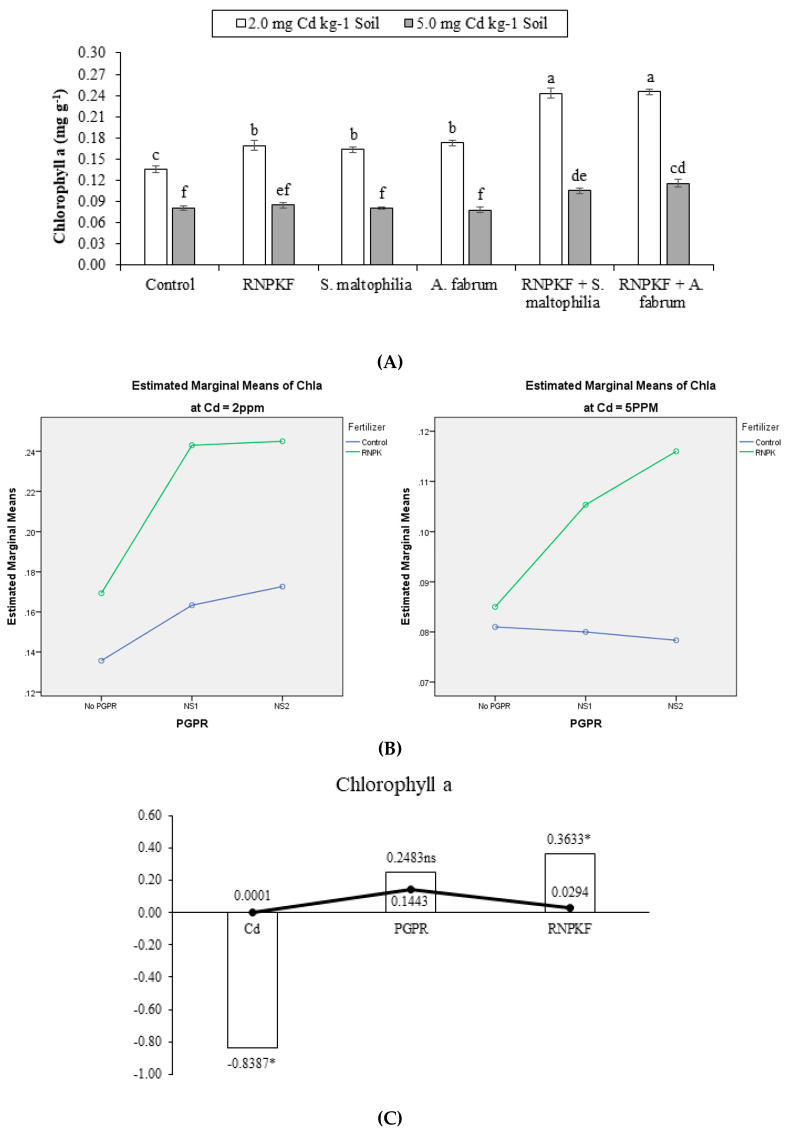
Effect of rhizobacteria with and without recommended NPK fertilizer on chlorophyll a (Chla) of bitter gourd under Cd (**A**). Interaction graph of PGPR (NS1 = *S. maltophilia*, NS2 = *A. fabrum*), RNPKF and Cd levels for Chla (mg g^−1^) (**B**). Pearson correlation of Chla with different levels of Cd, ACCD PGPR, and recommended NPK fertilizer where the *y*-axis represents the values of correlation (bars in the graph) and *x*-axis probability (lines in the graph) (**C**). * = *p* ≤ 0.05; ns = *p* > 0.05 non-significant.

Under Cd2 and Cd5, rhizobacteria, i.e., *A. fabrum*, *S. maltophilia*, and recommended NPK fertilizer showed a significantly (*p* ≤ 0.05) positive influence on the bitter gourd chlorophyll a content (Chla). Both interactive and main effects of PGPR and recommended NPK fertilizer were significant on Chla content at Cd2 and Cd5 (Figure 4A). In specific, PGPR × recommended NPK fertilizer has a significant ordinal interaction for Chla at Cd2 and Cd5 (Figure 4B). Treatments of recommended NPK fertilizer + *S. maltophilia*; recommended NPK fertilizer + *A. fabrum*; recommended NPK fertilizer; *S. maltophilia;* and *A. fabrum* responded positively at Cd2, so recommended NPK fertilizer is beneficial for Chla. It was noted that recommended NPK fertilizer + *S. maltophilia* and recommended NPK fertilizer + *A. fabrum* was significant for Chla at Cd5. Rhizobacteria i.e., *S. maltophilia* and *A. fabrum* sole inoculation showed similar response towards Chla in bitter gourd at Cd5. Correlation of Chla was negatively significant (−0.8387) at the different levels of Cd. Non-significant positive correlation (0.2483) was noted between PGPR and Chla, while significant positive correlation (0.3633) was recorded between Chla and recommended NPK fertilizer (Figure 4C). A significant increase of 78.5 and 50% in Chla was noted in recommended NPK fertilizer + *A. fabrum* compared to the control at Cd2 and Cd5, respectively.

### 2.5. Chlorophyll b Content in Bitter Gourd

**Figure 5:**
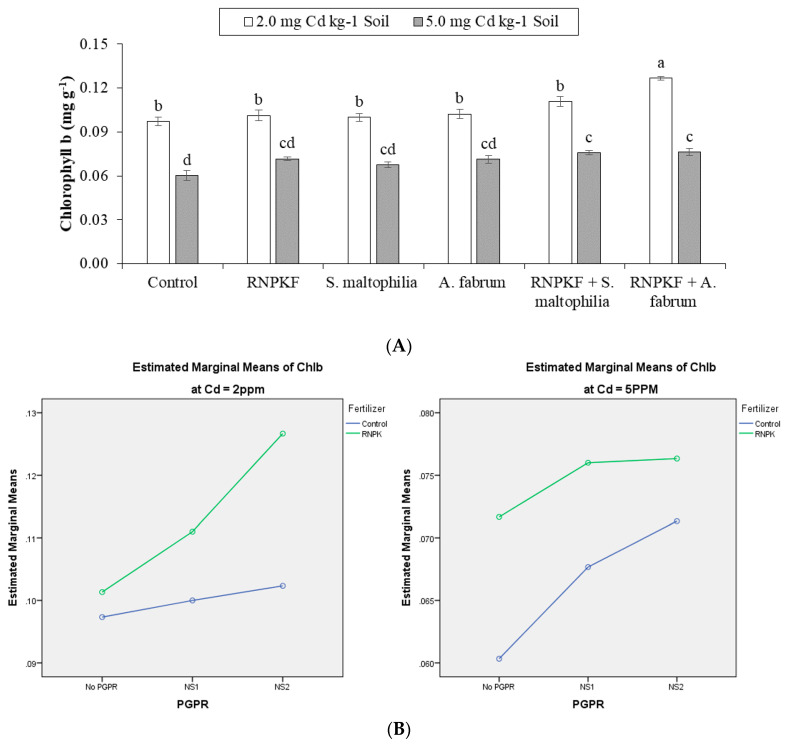

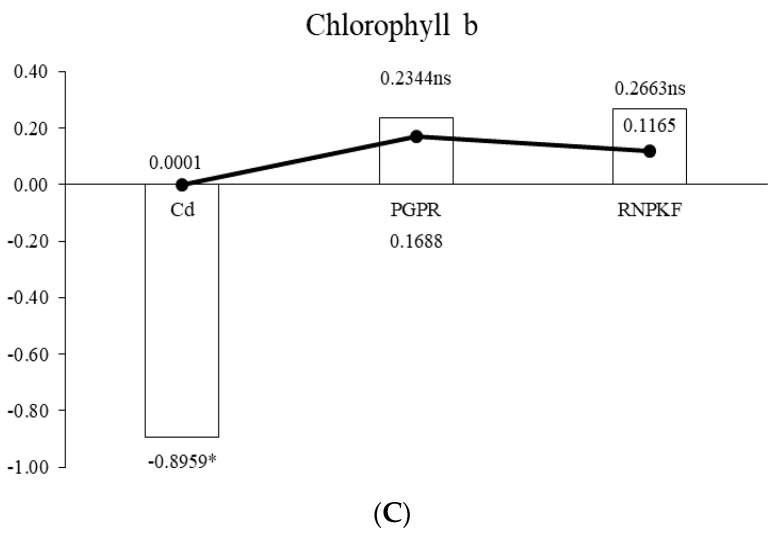
Effect of rhizobacteria with and without recommended NPK fertilizer on chlorophyll b (Chlb) of bitter gourd under Cd (**A**). Interaction graph of PGPR (NS1 = *S. maltophilia*, NS2 = A. Figure 1). (**B**). Pearson correlation of Chlb with different levels of Cd, ACCD PGPR, and recommended NPK fertilizer where the *y*-axis represents the values of correlation (bars in the graph) and *x*-axis probability (lines in the graph) (**C**). * = *p* ≤ 0.05; ns = *p* > 0.05 non-significant.

The effect of recommended NPK fertilizer and rhizobacteria (*S. maltophilia*, *A. fabrum*) remained significant (*p* ≤ 0.05) for the chlorophyll b content (Chlb) of bitter gourd under Cd2 and Cd5 (Figure 5A). The main effect of PGPR and recommended NPK fertilizer was significant at Cd2 and Cd5 but a significant ordinal interaction (PGPR × recommended NPK fertilizer) was noted only at Cd2 (Figure 5B). In addition, PGPR × recommended NPK fertilizer was non-significant but ordinal at Cd5 for Chlb. Application of recommended NPK fertilizer + *S. maltophilia* and recommended NPK fertilizer + *A. fabrum* showed similar responses towards Chlb at Cd5. Recommended NPK fertilizer + *A. fabrum* gave a significant increase comparative to the control for Chlb at Cd2. Inoculation of *S. maltophilia* and *A. fabrum* showed statistically alike effects for Chlb at Cd2 and Cd5. Correlation of Chlb was negatively significant (−0.8959) at different levels of Cd. Non-significant positive (0.2344) correlation was noted between PGPR and Chlb as well as between Chla and recommended NPK fertilizer (0.2663) (Figure 5C). In response to recommended NPK fertilizer + *A.fabrum,* Chlb was 30 and 33% higher in bitter gourd compared to the control at Cd2 and Cd5 respectively.

### 2.6. Total Chlorophyll Content in Bitter Gourd

**Figure 6:**
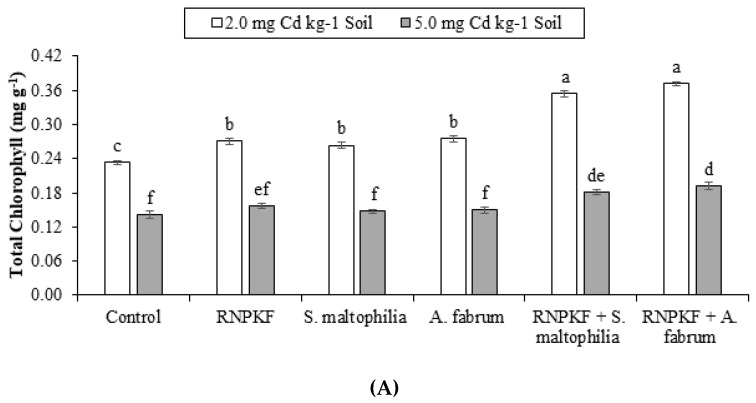

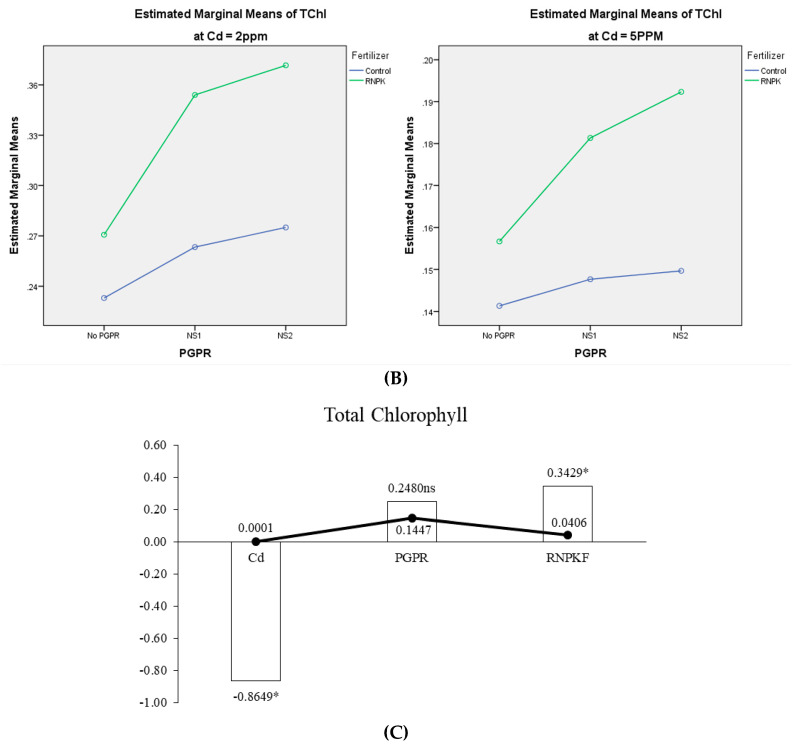
Effect of rhizobacteria with and without recommended NPK fertilizer on total chlorophyll (TChl) of bitter gourd under Cd (**A**). Interaction graph of PGPR (NS1 = *S. maltophilia*, NS2 = *A. fabrum*), RNPKF and Cd levels for TChl (mg g^−1^) (**B**). Pearson correlation of TChl with different levels of Cd, ACCD PGPR, and recommended NPK fertilizer where the *y*-axis represents the values of correlation (bars in the graph) and *x*-axis probability (lines in the graph) (**C**). * = *p* ≤ 0.05; ns = *p* > 0.05 non-significant.

Treatments of *S. maltophilia*, *A. fabrum*, and recommended NPK fertilizer under Cd2 and Cd5 remained significant (*p* ≤ 0.05) for the improvement in the total chlorophyll content (TChl) of bitter gourd. PGPR and recommended NPK fertilizer main effects were significant for enhancement in TChl at Cd2 and Cd5 (Figure 6A). Furthermore, interactions of PGPR and recommended NPK fertilizer differed significantly and ordinal for TChl only at Cd2. Non-significant but ordinal interaction (PGPR × recommended NPK fertilizer) was observed at Cd5 (Figure 6B) for TChl. Recommended NPK fertilizer + *S. maltophilia* and recommended NPK fertilizer + *A. fabrum* caused significant enhancement in TChl compared to the controls at Cd2 and Cd5. Results confirmed that recommended NPK fertilizer, *S. maltophilia*, and *A. fabrum* differed significantly at Cd2 but induced no significant change over control at Cd5 toxicity for TChl. Among *S. maltophilia* and *A. fabrum* treatments, TChl remained the same at Cd2 and Cd5. Inoculation of *S. maltophilia* and *A. fabrum* showed statistically alike effects for Chlb at Cd2 and Cd5. Correlation of Chlb was negative significant (−0.8649) at the different levels of Cd. Non-significant positive correlation (0.2480) was noted between PGPR and Chlb as well as between Chla and recommended NPK fertilizer (0.3429) (Figure 6C). The highest increases of 61 and 36% in TChl was observed in recommended NPK fertilizer + *A. fabrum* at Cd2 and Cd5 respectively.

### 2.7. Cadmium in Bitter Gourd

**Figure 7:**
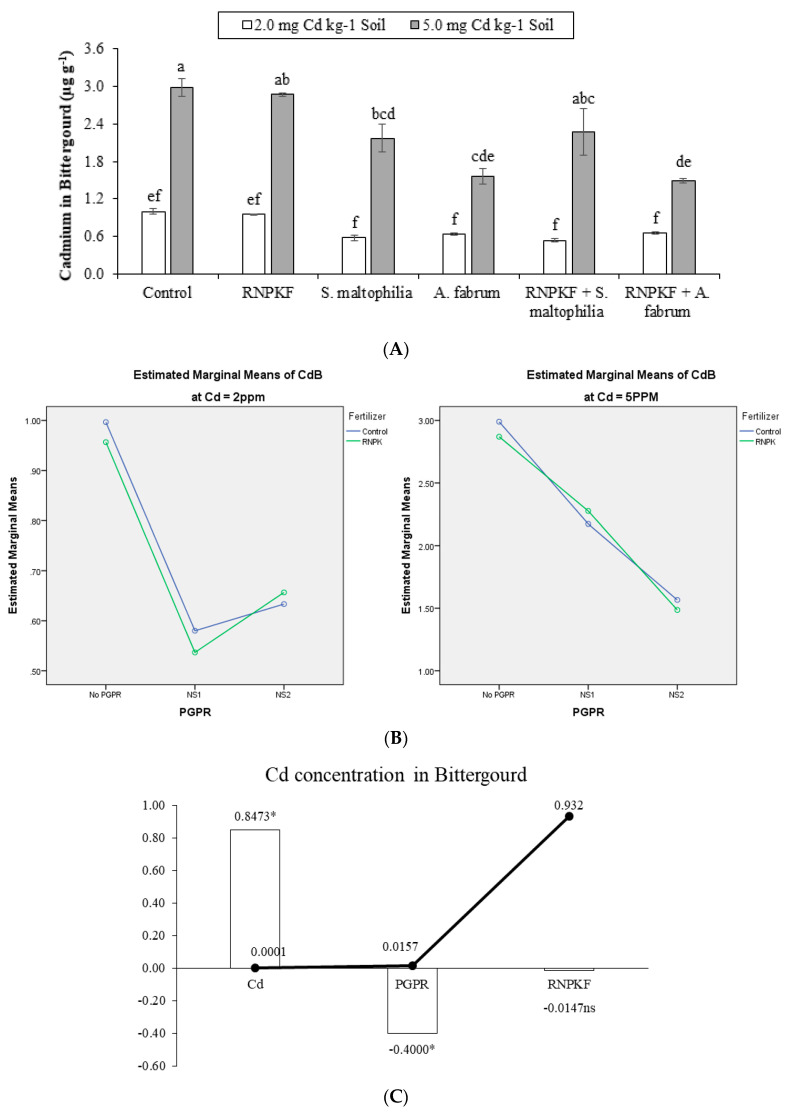
Effect of rhizobacteria with and without recommended NPK fertilizer on Cd in bitter gourd (CdB) under Cd (**A**). Interaction graph of PGPR (NS1 = *S. maltophilia*, NS2 = *A. fabrum*), RNPKF, and Cd levels for CdB (µg g^−1^) (**B**). Pearson correlation of CdB with different levels of Cd, ACCD PGPR, and recommended NPK fertilizer where the *y*-axis represents the values of correlation (bars in the graph) and *x*-axis probability (lines in the graph) (**C**). * = *p* ≤ 0.05; ns = *p* > 0.05 non-significant.

Cadmium concentration in bitter gourd (CdB) was affected significantly due to the application of *S. maltophilia*, *A. fabrum*, and recommended NPK fertilizer under Cd2 and Cd5. The main effect of PGPR was significant for CdB at Cd2 and Cd5 (Figure 7A). However, ordinal interaction was recorded between recommended NPK fertilizer and PGPR at Cd2 and Cd5 (Figure 7B) for CdB. Both recommended NPK fertilizer + *A. fabrum*, and *A. fabrum* were equally efficacious towards the reduction in bitter gourd Cd at Cd5. However, it was noted that recommended NPK fertilizer + *A. fabrum*, and *A. fabrum* showed a negative significant effect at Cd5 for CdB, as compared to control plants. Responses to all the treatments were statistically similar for CdB at Cd2. Correlation of CdB was positively significant (0.8473) at the different levels of Cd. Significant negative correlation (−0.4000) was noted between PGPR and CdB, while a non-significant negative (−0.0147) correlation was recorded between CdB and recommended NPK fertilizer (Figure 7C). A significant reduction of 50% in CdB was observed in recommended NPK fertilizer + *A. fabrum* at Cd5.

### 2.8. Cadmium in Soil

**Figure 8:**
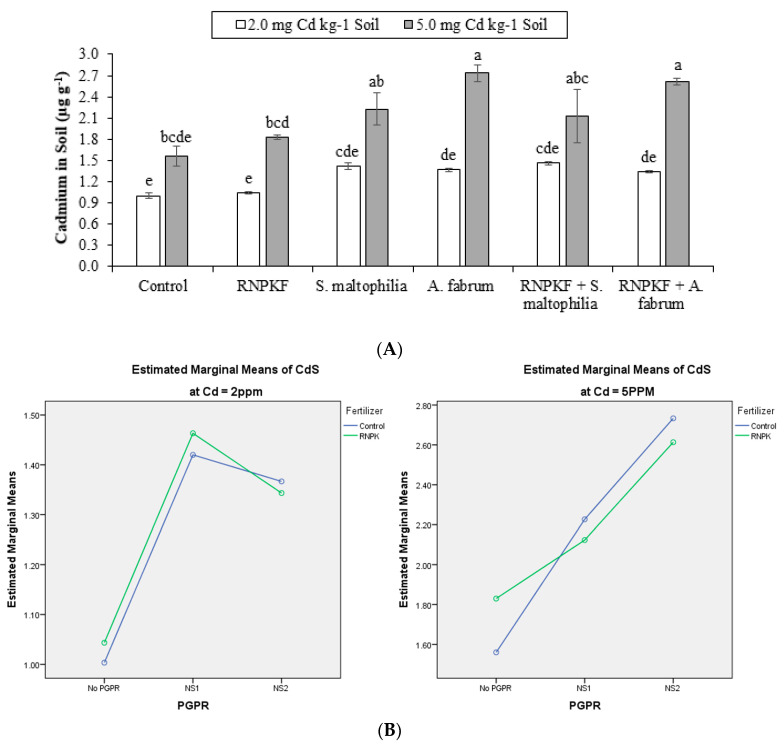

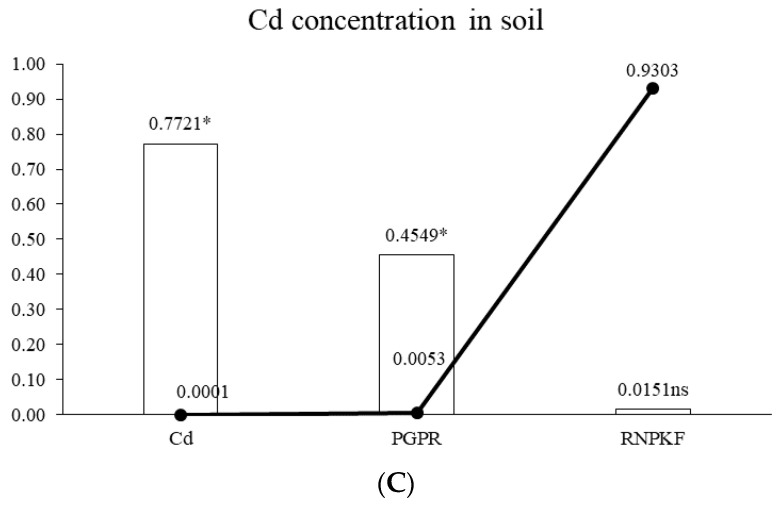
Effect of rhizobacteria with and without recommended NPK fertilizer on Cd in soil (CdS) under Cd (**A**). Interaction graph of PGPR (NS1 = *S. maltophilia*, NS2 = *A. fabrum*), RNPKF, and Cd levels for CdS (µg g^−1^) (**B**). Pearson correlation of CdS with different levels of Cd, ACCD PGPR, and recommended NPK fertilizer where the *y*-axis represents the values of correlation (bars in the graph) and *x*-axis probability (lines in the graph) (**C**). * = *p* ≤ 0.05; ns = *p* > 0.05 non-significant.

For cadmium concentration in soil (CdS), the responses of *S. maltophilia*, *A. fabrum*, and recommended NPK fertilizer were significant (*p* ≤ 0.05) (Figure 8A). Only PGPR has a significant ME, while interaction (PGPR × recommended NPK fertilizer) was ordinal for CdS at Cd2 and Cd5 (Figure 8B). Recommended NPK fertilizer + *A. fabrum,* and *A. fabrum* remained statistically alike each other for soil cadmium concentration at Cd5. However, positive responses of recommended NPK fertilizer + *A. fabrum*, and *A. fabrum* were noted for CdS at Cd5 compared to control plants. No significant fluctuation was observed in soil CdS among all the treatments at Cd2. Correlation of CdS was positively significant (0.7721) at the different levels of Cd. A significant positive correlation (0.4549) was noted between PGPR and CdS, while a non-significant positive (0.0151) correlation was recorded between CdS and recommended NPK fertilizer (Figure 8C). However, the sole inoculation of *A. fabrum* caused 75% increase in CdS over control plants at Cd5.

## 3. Discussion

In the current study, a significant decline in growth attributes was observed in the control. It was noted that chlorophyll contents were also significantly low where no amendment was applied. This reduction in growth was due to higher uptake of Cd in bitter gourd plants. Higher stress-generating ethylene levels might be another reason for the significant decline in growth and chlorophyll contents in bitter gourd. Sanita di Toppi and Gabbrielli [39] argued that Cd after a safe limit usually disturbs nutrients’ homeostasis, which causes a decline in root and shoot length. According to Glick et al. [40] generation of stress ethylene under stress induced negative impacts on crops’ productivity. Heavy metals cause abnormal division of cell and chromosomal aberration [41] resulting in reduction of protochlorophyllide reductase activity, thus inducing chlorosis in leaves [42]. Matile et al. [43] argued that, under stress conditions, higher ethylene accumulation prompts decomposition of lipids in the cell wall. Due to lipid degradation in plants, ethylene comes into contact with the chlorophyllase (chlase) gene and activates it in the chloroplast. This chlorophyllase (chlase) degrades chlorophyll, resulting in chlorosis and poor photosynthesis. Furthermore, treatments with recommended NPK fertilizer + *A. fabrum* were significantly different from the sole application of control with recommended NPK fertilizer. Chlorophyll contents and growth attributes of bitter gourd were significantly improved through the addition of recommended NPK fertilizer + *A. fabrum*. The improvement in morphological attributes was due to less uptake of Cd and its immobilization in soil. In addition, less accumulation of ethylene by treatments with *A. fabrum,* and recommended NPK fertilizer + *A. fabrum* activities might be allied factors for bitter gourd yield and growth. Both PGPR can secrete ACC deaminase that cleaves ethylene. Findings of many scientists also support our above argument [12,21,29,34,35,44,45,46,47]. According to Glick et al. [40] ACC deaminase breaks ethylene into α-ketobutyrate and ammonia. This ammonia is utilized by PGPR as a source of nitrogen. Stress ethylene in roots released in the rhizosphere where the concentration of ethylene is low, thus stress generation via ethylene in plants is alleviated. It was also observed that root length was significantly high in *A. fabrum* when applied in combination with fertilizer under cadmium stress. Similarly, vine length was also improved where *A. fabrum* was inoculated in combination with inorganic fertilizer. The improvement in length was due to the secretion of indole acetic acid by *A. fabrum*. Tripathi et al. [48] reported that indole acetic acid production by PGPR is a major factor for the increase in root length [49]. Fuhrer [50] argued that the activity of ACC deaminase played an imperative role in the enhancement of the root growth. The improvement in the fresh weight of bitter gourd justified the efficacious functioning of recommended NPK fertilizer + *A. fabrum* under Cd toxicity in the current experiment. According to Danish and Zafar-ul-Hye [34], inoculation of PGPR increased the uptake of nutrients and water in plants. The improvement in potassium [51] and water uptake played an efficacious role in the enhancement of fresh weigh and yield of crops. Application of recommended NPK fertilizer + *A. fabrum* significantly immobilized Cd in soil at low and high levels of Cd. Therefore, Cd concentration was high in soils where *A. fabrum and* recommended NPK fertilizer are present. However, the low concentration of Cd in the control indicates a significant intake of Cd in bitter gourd. *A. fabrum* [52] *and S. maltophilia* [53] have the potential to produce siderophores which make stable complexes of Cd in soil, thus immobilizing Cd in soil [54]. Deficiency of Fe due to its antagonistic relation with Cd uptake in plants also caused chlorosis in leaves [15,19,20]. Improvement in chlorophyll contents validated the lower uptake of Cd in the current experiment. Wu et al. [55] also suggested that peptides are capable of binding metals and decreasing metal phyto-availability in soil. The mobilization of nutrients is subjected to activation of root-proton efflux in the cortex of roots due to PGPR [56]. The findings of Belimov et al. [57] also supported the above argument of better nutrients uptake via ACC deaminase PGPR. Ardakani et al. [58] reported a significant enhancement in rice yield when PGPR i.e., *Azospirillum lipoferum* and *Pseudomonas fluorescens* were inoculated with fertilizer. The findings of Danish et al. [21] also supported our results regarding the alleviation of heavy-metals stress by ACC deaminase-producing rhizobacteria.

## 4. Materials and Methods

### 4.1. Site of Experiment

The pot experiment was carried out in 2016 in the research area of the Department of Soil Science, Bahauddin Zakariya University, Multan, Pakistan, on bitter gourd (Faisalabad long cv.) plants grown in cadmium-contaminated soil. The soil was characterized as dark brown and saline with the JAKHAR soil series [45]. The physiochemical property vary with land use [57,58,59] and are provided in Table 1.

### 4.2. Collection of Bacterial Strains and Broth

The bacterial strains were taken from the Laboratory of Soil Microbiology Department of Soil Science. PGPRs, i.e., *S. maltophilia* and *A. fabrum* were able to survive over 5.0 ppm Cd toxicity [12]. The PGPRs broth was kept over 25 °C for 48 h and uniform optical density was read using a spectrophotometer. The characteristics of PGPR were (Table 2)

### 4.3. Cadmium Toxicity and Plants Treatments

Analytical grade salt of cadmium chloride was used to introduce toxicity of Cd in soil [12]. There were two level of Cd. The 0.1% HgCl_2_ were used for seeds sterilization. The seeds were placed in the solution for 5 min [59]. 1 mL inoculum was used for seeds inoculation for attaching the bacteria to seeds, and the sugar peat and clay were also used with a 1:2:6 ratio for 100 g seeds and then the seeds were placed overnight. Four seeds were manually sown per pot. After germination, three seedlings were maintained via thinning. On a weight basis, 65% field capacity was maintained throughout the experiment. To fulfill the demand of the crops, nutrients were applied at the rate of 187 N, 75 P, and 225 K (kg ha^−1^) by using urea, K_2_HPO_4_, and K_2_SO_4_.

There were six treatments with three replications following two factorials with completely randomized design (CRD). The treatments included: control (without NPK and bacterial strains); recommended NPK fertilizer (RNPKF); *Stenotrophomonas maltophilia*; *Agrobacterium fabrum*; RNPKF + *S. maltophilia;* and recommended NPK fertilizer + *A. fabrum*. All the treatments were applied at two levels of Cd, i.e., 2 and 5 mg kg^−1^ soil (Cd2 and Cd5).

### 4.4. Analysis of Morphological Attributes and Chlorophyll Contents

At the fruit maturity, harvesting was done. Morphological attributes were noted manually. Fresh weight of fruit was measured using a weight balance. Vine and root length were recorded after 95 days of sowing.

### 4.5. Chlorophyll Contents

The chlorophyll a, chlorophyll b, and total chlorophyll contents were examined by following Aron [57].

$$\mathrm{Chloropyll}\;a\;\left({\mathrm{mg}\;g}^{-1}\;f.\mathrm{wt}.\right)=\;\frac{12.7\;\left(\mathrm{OD}\;663\right)-2.69\;\left(\mathrm{OD}\;645\right)V}{1000\;\left(W\right)}$$

$$\mathrm{Chloropyll}\;a\;\left({\mathrm{mg}\;g}^{-1}\;f.\mathrm{wt}.\right)=\;\frac{22.9\;\left(\mathrm{OD}645\right)-4.68\;\left(\mathrm{OD}\;663\right)V}{1000\;\left(W\right)}$$

V = final volume made; W = gram of fresh leaf sample, and f. wt. = fresh weight. Total chlorophyll was calculated by the addition of chlorophyll a and chlorophyll b.

### 4.6. Cadmium Analyses

With the help of diacid mixture (nitric and perchloric acid in 2:1 ratio), the tissues of the plant were digested. During the digestion, the temperature was gradually increased and maintained up to 280 °C [60]. After filtration, the sample was run on an atomic absorption spectrophotometer for the analyses of Cd.

### 4.7. Statistical Analysis

One-way ANOVA was used to assess the effects of treatments. Two-factors ANOVA was conducted separately to compare PGPR and RNPK interactions with Cd2 and Cd5 by using SPSS 18.0 statistical software (SPSS Inc. Released 2009. PASW Statistics for Windows, Version 18.0. Chicago, IL, USA). Treatments comparison was computed at *p* ≤ 0.05 by Tukey’s test using standards statistical techniques [61].

## 5. Conclusions

In conclusion, ACC deaminase-producing rhizobacteria *A. fabrum* has a higher potential over *S. maltophilia* to decrease the Cd uptake in bitter gourd. Less uptake of Cd in bitter gourd improved the vine length, root length, and fresh weight attributes. The combined application of recommended NPK fertilizer and *A. fabrum* can increase the growth and chlorophyll contents of bitter gourd plants grown under Cd stress. Application of *S. maltophilia* was also efficacious in improving the growth traits of bitter gourd under Cd stress. However, *A. fabrum* with recommended NPK fertilizer is suggested to immobilize Cd in soil and to improve the bitter gourd yield in Cd-contaminated soils.

## Figures and Tables

**Table 1:** Characteristics of experimental soil.

Attributes	Units	Values
Sand	%	40
Silt	%	40
Clay	%	20
Textural Class	-	Loam
Saturation Percentage	%	40
pH*s*	-	8.05
EC*e*	dS m^−1^	5.15
Available Phosphorus	mg kg^−1^	7.62
Extractable Potassium	mg kg^−1^	176

**Table 2:** Characteristics of plant growth-promoting rhizobacteria.

Characteristics	*A. fabrum*	*S. maltophilia*
P-Solubilization (μg/mL)	16.0	9.36
K-Solubilization (μg/mL)	25.04	12.35
IAA (with L-Tryptophan) (μg/mL)	50.20	21.83
IAA (without L-Tryptophan) (μg/mL)	2.01	1.09
ACCD activity (μmol α-ketobutyrate nmol mg^−1^ protein h^−1^)	340	145

## References

[1] Miniraj N., Prasanna K.P., Peter K.V., Kalloo G., Bergh B.O. (1993). Bitter gourd *Momordica* spp.. Genetic Improvement of Vegetable Plants.

[2] Liu X.R., Deng Z.Y., Fan Y.W., Li J., Liu Z.H. (2010). Mineral elements analysis of momordica charantiap seeds by ICP-AES and fatty acid profile identification of seed oil by GC-MS. Spectrosc. Spectr. Anal..

[3] Cao H., Sethumadhavan K., Grimm C.C., Ullah A.H.J. (2014). Characterization of a soluble phosphatidic acid phosphatase in bitter melon (*Momordica charantia*). PLoS ONE.

[4] GOP (2016). Fruits, Vegetables and Condiments: Statistics of Pakistan.

[5] Fang E.F., Ng T.B. (2016). Bitter Gourd (*Momordica charantia*) Oils. Essential Oils in Food Preservation, Flavor and Safety.

[6] Ahmad I., Akhtar M.J., Zahir Z.A., Naveed M., Mitter B., Sessitsch A. (2014). Cadmium-tolerant bacteria induce metal stress tolerance in cereals. Environ. Sci. Pollut. Res..

[7] Meena R.S., Kumar S., Datta R., Lal R., Vijayakumar V., Brtnicky M., Sharma M.P., Yadav G.S., Jhariya M.K., Jangir C.K. (2020). Impact of Agrochemicals on Soil Microbiota and Management: A Review. Land.

[8] Brtnicky M., Dokulilova T., Holatko J., Pecina V., Kintl A., Latal O., Vyhnanek T., Prichystalova J., Datta R. (2019). Long-Term Effects of Biochar-Based Organic Amendments on Soil Microbial Parameters. AGRONOMY-BASEL.

[9] Molaei A., Lakzian A., Datta R., Haghnia G., Astaraei A., Rasouli-Sadaghiani M., Ceccherini M.T. (2017). Impact of chlortetracycline and sulfapyridine antibiotics on soil enzyme activities. Int. Agrophysics.

[10] Molaei A., Lakzian A., Haghnia G., Astaraei A., Rasouli-Sadaghiani M., Ceccherini M.T., Datta R. (2017). Assessment of some cultural experimental methods to study the effects of antibiotics on microbial activities in a soil: An incubation study. PLoS ONE.

[11] Lazar V., Cernat R., Balotescu C., Cotar A., Coipan E., Cojocaru C. (2002). Correlation between multiple antibiotic resistance and heavy-metal tolerance among some E.coli strains isolated from polluted waters. Bacteriol. Virusol. Parazitol. Epidemiol..

[12] Zafar-ul-Hye M., Shahjahan A., Danish S., Abid M., Qayyum M.F. (2018). Mitigation of cadmium toxicity induced stress in wheat by ACC-deaminase containing PGPR isolated from cadmium polluted wheat rhizosphere. Pakistan J. Bot..

[13] Zafar-Ul-Hye M., Naeem M., Danish S., Fahad S., Datta R., Abbas M., Rahi A.A., Brtnický M., Holatko J., Tarar Z.H. (2020). Alleviation of Cadmium Adverse Effects by Improving Nutrients Uptake in Bitter Gourd through Cadmium Tolerant Rhizobacteria. Environments.

[14] Lamoreaux R.J., Chaney W.R. (1978). The Effect of Cadmium on Net Photosynthesis, Transpiration, and Dark Respiration of Excised Silver Maple Leaves. Issue Physiol. Plant. Addit. Inf..

[15] Larbi A., Morales F., Abadia A., Gogorcena Y., Lucena J.J., Abadia J. (2002). Effects of Cd and Pb in sugar beet plants grown in nutrient solution: Induced Fe deficiency and growth inhibition. Funct. Plant Biol..

[16] Huang C.-Y., Bazzaz F.A., Vanderhoef L.N. (1974). The inhibition of soybean metabolism by cadmium and lead. J. Plant Physiol..

[17] Wallace A., Wallace G.A., Cha J.W. (1992). Some modifications in trace metal toxicities and deficiencies in plants resulting from interactions with other elements and chelating agents--the special case of iron. J. Plant Nutr..

[18] Abid M., Danish S., Zafar-ul-Hye M., Shaaban M., Iqbal M.M., Rehim A., Qayyum M.F., Naqqash M.N. (2017). Biochar increased photosynthetic and accessory pigments in tomato (*Solanum lycopersicum* L.) plants by reducing cadmium concentration under various irrigation waters. Environ. Sci. Pollut. Res..

[19] Greger M., Brammer E., Lindberg S., Larsson G., Idestam-Almquist J. (1991). Uptake and Physiological Effects of Cadmium in Sugar Beet (*Beta vulgaris*) Related to Mineral Provision. J. Exp. Bot..

[20] Dong J., Wu F., Zhang G. (2006). Influence of cadmium on antioxidant capacity and four microelement concentrations in tomato seedlings (*Lycopersicon esculentum*). Chemosphere.

[21] Danish S., Kiran S., Fahad S., Ahmad N., Ali M.A., Tahir F.A., Rasheed M.K., Shahzad K., Li X., Wang D. (2019). Alleviation of chromium toxicity in maize by Fe fortification and chromium tolerant ACC deaminase producing plant growth promoting rhizobacteria. Ecotoxicol. Environ. Saf..

[22] Pandey S., Ghosh P.K., Ghosh S., De T.K., Maiti T.K. (2013). Role of heavy metal resistant *Ochrobactrum* sp. and *Bacillus* spp. strains in bioremediation of a rice cultivar and their PGPR like activities. J. Microbiol..

[23] Khan A.L., Lee I.-J. (2013). Endophytic Penicillium funiculosum LHL06 secretes gibberellin that reprograms *Glycine max* L. growth during copper stress. BMC Plant Biol..

[24] Glick B., Penrose D., Li J. (1998). A Model For the Lowering of Plant Ethylene Concentrations by Plant Growth-promoting Bacteria. J. Theor. Biol..

[25] Skirycz A., Claeys H., de Bodt S., Oikawa A., Shinoda S., Andriankaja M., Maleux K., Eloy N.B., Coppens F., Yoo S.D. (2011). Pause-and-stop: The effects of osmotic stress on cell proliferation during early leaf development in Arabidopsis and a role for ethylene signaling in cell cycle arrest. Plant Cell.

[26] He C.J., Morgan P.W., Drew M.C. (1996). Transduction of an ethylene signal is required for cell death and lysis in the root cortex of maize during aerenchyma formation induced by hypoxia. Plant Physiol..

[27] Garnier L., Simon-Plas F., Thuleau P., Agnel J.P., Blein J.P., Ranjeva R., Montillet J.L. (2006). Cadmium affects tobacco cells by a series of three waves of reactive oxygen species that contribute to cytotoxicity. Plant Cell Environ..

[28] Das K., Roychoudhury A. (2014). Reactive oxygen species (ROS) and response of antioxidants as ROS-scavengers during environmental stress in plants. Front. Environ. Sci..

[29] Zafar-ul-Hye M., Tahzeeb-ul-Hassan M., Abid M., Fahad S., Brtnicky M., Dokulilova T., Datta R., Danish S. (2020). Potential role of compost mixed biochar with rhizobacteria in mitigating lead toxicity in spinach. Sci. Rep..

[30] Kochar M., Upadhyay A., Srivastava S. (2011). Indole-3-acetic acid biosynthesis in the biocontrol strain Pseudomonas fluorescens Psd and plant growth regulation by hormone overexpression. Res. Microbiol..

[31] Parewa H.P., Meena V.S., Jain L.K., Choudhary A. (2018). Sustainable crop production and soil health management through plant growth-promoting rhizobacteria. Role of Rhizospheric Microbes in Soil: Stress Management and Agricultural Sustainability.

[32] Adnan M., Fahad S., Zamin M., Shah S., Mian I.A., Danish S., Zafar-ul-Hye M., Battaglia M.L., Naz R.M.M., Saeed B. (2020). Coupling phosphate-solubilizing bacteria with phosphorus supplements improve maize phosphorus acquisition and growth under lime induced salinity stress. Plants.

[33] Wahid F., Fahad S., Danish S., Adnan M., Yue Z., Saud S., Siddiqui M.H., Brtnicky M., Hammerschmiedt T., Datta R. (2020). Sustainable Management with Mycorrhizae and Phosphate Solubilizing Bacteria for Enhanced Phosphorus Uptake in Calcareous Soils. Agriculture.

[34] Danish S., Zafar-ul-Hye M. (2019). Co-application of ACC-deaminase producing PGPR and timber-waste biochar improves pigments formation, growth and yield of wheat under drought stress. Sci. Rep..

[35] Zafar-ul-Hye M., Danish S., Abbas M., Ahmad M., Munir T.M. (2019). ACC deaminase producing PGPR Bacillus amyloliquefaciens and agrobacterium fabrum along with biochar improve wheat productivity under drought stress. Agronomy.

[36] Danish S., Zafar-Ul-Hye M., Hussain S., Riaz M., Qayyum M.F. (2020). Mitigation of drought stress in maize through inoculation with drought tolerant ACC deaminase containing PGPR under axenic conditions. Pakistan J. Bot..

[37] Danish S., Zafar-ul-Hye M., Fahad S., Saud S., Brtnicky M., Hammerschmiedt T., Datta R. (2020). Drought Stress Alleviation by ACC Deaminase Producing Achromobacter xylosoxidans and Enterobacter cloacae, with and without Timber Waste Biochar in Maize. Sustainability.

[38] Zafar-Ul-hye M., Farooq U., Danish S., Hussain S., Shaaban M., Qayyum M.F., Rehim A. (2020). Bacillus amyloliquefaciens and alcaligenes faecalis with biogas slurry improved maize growth and yield in saline-sodic field. Pakistan J. Bot..

[39] Sanita di Toppi L., Gabbrielli R. (1999). Response to cadmium in higher plants. Environ. Exp. Bot..

[40] Glick B.R., Patten C.L., Holguin G., Penrose D.M. (1999). Biochemical and Genetic Mechanisms Used by Plant Growth Promoting Bacteria.

[41] Ouzounidou G., Ciamporova M., Moustakas M., Karataglis S. (1995). Responses of Maize (*Zea-Mays* L) Plants to Copper Stress.1. Growth, Mineral-Content and Ultrastructure of Roots. Environ. Exp. Bot..

[42] De Filippis L.F., Hampp R., Ziegler H. (1981). The effects of sublethal concentrations of zinc, cadmium and mercury on Euglena. Arch. Microbiol..

[43] Matile P., Schellenberg M., Vicentini F. (1997). Planta Localization of chlorophyllase in the chloroplast envelope. Planta.

[44] Danish S., Zafar-ul-Hye M., Hussain M., Shaaban M., Núñez-delgado A. (2019). Rhizobacteria with ACC-Deaminase Activity Improve Nutrient Uptake, Chlorophyll Contents and Early Seedling Growth of Wheat under PEG- Induced Osmotic Stress. Int. J. Agric. Biol..

[45] Ahmed N., Ahsen S., Ali M.A., Hussain M.B., Hussain S.B., Rasheed M.K., Butt B., Irshad I., Danish S. (2020). Rhizobacteria and silicon synergy modulates the growth, nutrition and yield of mungbean under saline soil. Pakistan J. Bot..

[46] Danish S., Zafar-ul-Hye M. (2020). Combined role of ACC deaminase producing bacteria and biochar on cereals productivity under drought. Phyton (B. Aires).

[47] Danish S., Zafar-ul-Hye M., Mohsin F., Hussain M. (2020). ACC-deaminase producing plant growth promoting rhizobacteria and biochar mitigate adverse effects of drought stress on maize growth. PLoS ONE.

[48] Tripathi M., Munot H.P., Shouche Y., Meyer J.M., Goel R. (2005). Isolation and functional characterization of siderophore-producing lead- and cadmium-resistant Pseudomonas putida KNP9. Curr. Microbiol..

[49] Burd G.I., Dixon D.G., Glick B.R. (1998). A plant growth-promoting bacterium that decreases nickel toxicity in seedlings. Appl. Environ. Microbiol..

[50] Fuhrer J. (1982). Ethylene Biosynthesis and Cadmium Toxicity in Leaf Tissue of Beans (*Phaseolus vulgaris* L.). Plant Physiol..

[51] Gilani M., Danish S., Ahmed N., Rahi A.A., Akrem A., Younis U., Irshad I., Iqbal R.K. (2020). Mitigation of drought stress in spinach using individual and combined applications of salicylic acid and potassium. Pakistan J. Bot..

[52] Baude J., Vial L., Villard C., Campillo T., Lavire C., Nesme X., Hommais F. (2016). Coordinated regulation of species-specific hydroxycinnamic acid degradation and siderophore biosynthesis pathways in Agrobacterium fabrum. Appl. Environ. Microbiol..

[53] Gopi K., Jinal H.N., Prittesh P., Kartik V.P., Amaresan N. (2020). Effect of copper-resistant Stenotrophomonas maltophilia on maize (*Zea mays*) growth, physiological properties, and copper accumulation: Potential for phytoremediation into biofortification. Int. J. Phytoremediation.

[54] Rajkumar M., Sandhya S., Prasad M.N.V., Freitas H. (2012). Perspectives of plant-associated microbes in heavy metal phytoremediation. Biotechnol. Adv..

[55] Wu C.H., Wood T.K., Mulchandani A., Chen W. (2006). Engineering plant-microbe symbiosis for rhizoremediation of heavy metals. Appl. Environ. Microbiol..

[56] Safronova V.I., Stepanok V.V., Engqvist G.L., Alekseyev Y.V., Belimov A.A. (2006). Root-associated bacteria containing 1-aminocyclopropane-1-carboxylate deaminase improve growth and nutrient uptake by pea genotypes cultivated in cadmium supplemented soil. Biol. Fertil. Soils.

[57] Belimov A.A., Safronova V.I., Sergeyeva T.A., Egorova T.N., Matveyeva V.A., Tsyganov V.E., Borisov A.Y., Tikhonovich I.A., Kluge C., Preisfeld A. (2001). Characterization of plant growth promoting rhizobacteria isolated from polluted soils and containing 1-aminocyclopropane-1-carboxylate deaminase. Can. J. Microbiol..

[58] Ardakani M.R., Khorshidi Y.R., Ramezanpour M.R., Khavazi K., Zargari K. (2011). Response of Yield and Yield Components of Rice (*Oryza sativa* L.) to Pseudomonas flouresence and Azospirillum lipoferum under Different Nitrogen Levels. Am. J. Agric. Environ. Sci..

[59] Sadiq A., Ali B. (2013). Growth and yield enhancement of *Triticum aestivum* L. by rhizobacteria isolated from agronomic plants. Aust. J. Crop Sci..

[60] Chapman H.D., Pratt P.F. (1961). Methods of Analysis for Soils, Plants and Water.

[61] Steel R.G., Torrie J.H., Dickey D.A. (1997). Principles and Procedures of Statistics: A Biometrical Approach.

